# A Mobile Phone-Based Health Coaching Intervention for Weight Loss and Blood Pressure Reduction in a National Payer Population: A Retrospective Study

**DOI:** 10.2196/mhealth.7591

**Published:** 2017-06-08

**Authors:** Alice Yuqing Mao, Connie Chen, Candy Magana, Karla Caballero Barajas, J Nwando Olayiwola

**Affiliations:** ^1^ School of Medicine University of California San Francisco San Francisco, CA United States; ^2^ Department of Medicine Stanford University Palo Alto, CA United States; ^3^ RubiconMD New York, NY United States; ^4^ Vida Health Inc Mountain View, CA United States; ^5^ Department of Family & Community Medicine University of California San Francisco San Francisco, CA United States

**Keywords:** digital health coaching, overweight, obesity, mobile health, weight, blood pressure

## Abstract

**Background:**

The prevalence of obesity and associated metabolic conditions continue to be challenging and costly to address for health care systems; 71% of American adults were overweight, with 35% of men and 40% of women diagnosed with obesity in 2014. Digital health coaching is an innovative approach to decreasing the barriers of cost and accessibility of receiving health coaching for the prevention and management of chronic disease in overweight or obese individuals.

**Objective:**

To evaluate the early impact of a mobile phone-based health coaching service on weight loss and blood pressure management in a commercially insured population.

**Methods:**

This was a retrospective study using existing registry data from a pilot commercial collaboration between Vida Health and a large national insurance provider, which enrolled adult members who were overweight (body mass index >25 kg/m2) and able to engage in a mobile phone-based coaching intervention. Participants received 4 months of intensive health coaching via live video, phone, and text message through the Vida Health app. Participants were also provided with a wireless scale, pedometer, and blood pressure cuff. Of the 1012 enrolled, 763 (75.40%) participants had an initial weight upon enrollment and final weight between 3 and 5 months from enrollment; they served as our intervention group. There were 73 participants out of the 1012 (7.21%) who had weight data 4 months prior to and after Vida coaching, who served as the matched-pair control group.

**Results:**

Participants in the intervention group lost an average of 3.23% total body weight (TBW) at 4 months of coaching and 28.6% (218/763) intervention participants achieved a clinically significant weight loss of 5% or more of TBW, with an average of 9.46% weight loss in this cohort. In the matched-pair control group, participants gained on average 1.81% TBW in 4 months without Vida coaching and lost, on average, 2.47% TBW after 4 months of Vida coaching, demonstrating a statistically significant difference of 4.28% in mean percentage weight change (P<.001). Among 151 intervention participants with blood pressure data, 112 (74.2%) had a baseline blood pressure that was above the goal (systolic blood pressure >120 mmHg); 55 out of 112 (49.1%) participants improved their blood pressure at 4 months by an entire hypertensive stage—as defined by the Seventh Report of the Joint National Committee on Prevention, Detection, Evaluation, and Treatment of High Blood Pressure.

**Conclusions:**

Mobile phone app-based health coaching interventions can be an acceptable and effective means to promote weight loss and improve blood pressure management in overweight or obese individuals. Given the ubiquity of mobile phones, digital health coaching may be an innovative solution to decreasing barriers of access to much-needed weight management interventions for obesity.

## Introduction

The prevalence of obesity and associated metabolic conditions continue to be challenging and costly to address for health care systems; 71% of American adults were overweight, with 35% of men and 40% of women diagnosed with obesity in 2014 [1-5]. Being overweight and obese increases the risk of cardiometabolic disease and overall mortality, with the prevalence of hypertension and type 2 diabetes highly correlated to increasing weight [6]. Weight loss as modest as 5% total body weight in people with a body mass index (BMI) over 25 kg/m^2^ can significantly improve glycosylated hemoglobin, blood pressure, and hyperlipidemia, and can reduce the progression of hypertension and diabetes [7-9]. The United States Preventive Task Force recently updated its recommendations to formally include intensive, multicomponent interventions for weight loss in patients with a BMI ≥ 30 kg/m^2^, citing evidence showing its benefits in cardiovascular risk reduction [10].

With primary care clinicians in increasingly short supply and overwhelmed by burdens of the preventative and chronic care needs of their patient panel [11,12], numerous health care providers have sought to use trained health coaches to provide cost-effective, culturally sensitive behavioral counseling for weight loss and chronic disease management. There is compelling evidence that health coach-led programs can indeed significantly improve the control of metabolic risk factors [13-15]. Despite provisions in the US Affordable Care Act of mandated free obesity counseling to those who qualify and availability of robust evidence around behavior change programs for weight loss, uptake of behavioral-counseling weight loss programs has been limited. This is often due to difficulties with attending in-person sessions—offered by most programs—caused by wide-ranging issues such as transportation, childcare, work coverage, and limited availability of such programs, especially in rural settings [16].

In addition to decreasing the barriers of cost and accessibility for receiving health coaching, digital health coaching provided remotely through an Internet-connected device has been shown to be an effective approach for the prevention and management of cardiometabolic risk factors in overweight and diabetic individuals [17,18]. Given that 92% of US adults own a basic-feature mobile phone and 68% own an advanced-feature mobile phone (ie, iPhone or Android) [19], mobile devices may offer a novel continuous engagement portal for delivery of health coaching and intensive behavioral interventions for weight loss [20]. In addition to containing an array of sensors relevant to core aspects of behavior change programs (eg, pedometers to track steps, cameras to record food intake, audio and live video to communicate with a health coach), the vast majority of mobile phones are carried by users for the majority of their waking day, with users engaging with apps on their mobile devices for 3 hours and 5 minutes per day, on average [21].

While studies of Web- [17,18] and short message service (SMS) text message-based [22,23] coaching interventions have demonstrated positive results, the few number of studies examining mobile phone-based health coaching are limited either by sample size, a focus on the impact of self-monitoring rather than coaching, or a lack of clinical results beyond self-reported weight loss [24,25]. Thus, given the limited published research to date evaluating mobile phone-based coaching interventions for weight loss and cardiovascular risk reduction, the authors share here retrospective registry data from a pilot, commercial, mobile phone-based health-coaching program. The program was offered by a large national commercial insurance company to its fully insured members in the US states of Wisconsin, Georgia, and Colorado who were overweight and self-reported diagnoses of obesity, hypertension, prediabetes, dyslipidemia, or diabetes.

## Methods

### Overview

This was a retrospective study using existing registry data from a pilot commercial collaboration between Vida Health and a large insurance provider. Interested members under this insurance provider were enrolled starting in November 2015 in a commercial program using a mobile phone-based, digital health-coaching app, Vida Health (described below). The coaching platform was used to manage cardiometabolic conditions such as obesity, hypertension, hyperlipidemia, and prediabetes. This study has received Institutional Review Board (IRB) exemption from the University of California, San Francisco (UCSF), IRB (IRB No. 16-19903).

### Participants

The registry included adults over the age of 18 years who were fully insured members in the US states of Wisconsin, Georgia, and Colorado and who were overweight—BMI >25 kg/m^2^. Participants had to be English speaking and own an advanced-feature mobile phone—iPhone or Android—to ensure they were able to engage in the Vida Health coaching program. Participants were recruited from an email campaign by their insurance provider offering the free program for any current member who had a BMI >25 kg/m^2^. In order not to bias the sample with individuals who would have achieved weight loss and other health goals regardless of whether or not they worked with a coach, the barrier to entry into the program was kept at a minimum—simply replying to a Web form in an email invite—with minimal exclusion criteria, including not owning a mobile phone and having type 1 diabetes. Clinical data elements used for enrollment, including weight, height, and medical history, were based on self-reported responses. Invitations to the program were sent randomly to members in eligible geographies and the first 1000 participants were included in this pilot program.

### Intervention

Enrollment for the Vida Health program started November 2015. Prior to starting the Vida Health program, accepted members were sent a package containing a Bluetooth-connected pedometer and wireless scale. Those members with known hypertension also received a Bluetooth-enabled blood pressure cuff. Participants received instructions on how to synchronize their wireless devices for passive data collection through the Vida app.

After installing the Vida app, participants were asked to complete an onboarding survey regarding their baseline health behaviors, past medical history, self-selected personality preferences (ie, if they might benefit more from a “cheerleader”- or “drill sergeant”-like coach), and general availability. Based on these attributes, participants were matched with a short list of Vida-recommended coaches from which they could select their own ongoing health coach. Health coaches were professional licensed nutritionists, physical therapists, and social workers who are certified to provide health coaching and were additionally trained by Vida once employed in the Vida network.

The first 4 months of the Vida program consisted of an intensive *active coaching* phase followed by 8 months of *maintenance* coaching. During the active coaching phase, participants had regular consults—video chats or phone calls through the Vida app—with their coaches ranging from weekly to monthly in frequency. Different coaching frequencies were recommended for each participant based on the coach’s assessment of participants’ needs and availability. Participants were encouraged to weigh in on a weekly basis on their wireless scales. During this time period, coaches worked with participants to set personalized health goals around healthy nutrition, physical activity, stress management, and medication adherence, carefully tailored to advance participants’ weight loss goals. In between consults, coaches communicated with their clients via secure text messaging in the Vida app, providing daily accountability through quick reminders about clients’ personal goals, motivation through inspirational content, and education through easily understandable pieces of content.

In addition to data passively collected by the wireless scales, pedometers, and blood pressure cuffs provided to participants, members were also asked to enter their activity (ie, steps, exercise, food intake, sleep, and stress levels). Participants who had difficulty using or synchronizing the provided devices had the option of self-entering biometric data (ie, weight and blood pressure) directly into the Vida app. For the purposes of accurate analyses, we excluded metric points that were deemed to be unrealistic outliers that fell outside of the scope of clinical weight loss or the trend of the participant. Members were not paid for their participation. Program costs, including both the coaching service and associated hardware devices, were covered by their insurance provider.

### Measures

The primary outcome of the study was weight loss at 4 months as defined by percent change in total body weight (TBW) to normalize against a range of starting body weights. Participants were encouraged to weigh themselves weekly during the 4 months of the intensive coaching program. Weight data was primarily collected through the direct transmission of values via a Bluetooth scale provided as part of the intervention. Some participants chose to self-enter data due to technical difficulties setting up and using the Bluetooth scale. Change in TBW was calculated as the difference between the first weight since program enrollment and the last weight entered closest to the end of 4 months of coaching, with the caveat that the provided weight was recorded between 3 and 5 months after program enrollment.

Secondary outcomes included change in systolic blood pressure (SBP) after 4 months of intensive health coaching, as well as the change in number of participants in each hypertensive category (ie, normal, prehypertensive, type 1 hypertension, and type 2 hypertension) from the beginning of enrollment to after 4 months of coaching.

Satisfaction with the intervention was summarized using participant-reported ratings of the Vida Health app. Throughout the program, participants were asked, “How are we doing? Please help us improve your experience by taking a minute to leave feedback for your coach.” Participants could input text feedback and rate their experience on a scale of 0-10, with 10 being the highest rating.

### Control Group

Given that this study was based off of registry data from an existing pilot program, a designated control arm that did not receive coaching was not defined. However, there was a subset of individuals (n=73) enrolled in the program who had historic weight data from owning Bluetooth scales prior to starting the program. Using the Validic application programming interface, these historic weight data points were added to the Vida app database when individuals synchronized their devices as part of the program. These individuals’ historic weight data 4 months prior to starting the Vida program were used as a proxy for a matched-pair control group, as these individuals successfully enrolled and received coaching through the program, thereby eliminating any bias toward motivation.

### Analyses

Summary statistics describing the demographic characteristics are provided in Table 1, including age, gender, starting BMI, geographic state, and relevant self-reported medical history among enrolled and active participants who have recorded weight data.

Participant data were statistically analyzed using R version 3.3.3 (The R Foundation) using descriptive analysis and two-sided *t* tests to estimate the statistical difference between the preintervention and the postintervention measurements. Differences were compared in categorical and continuous data between the intervention and matched-pair control groups using chi-square and two-sample *t* tests, respectively. Absolute weight loss and change in percent TBW were calculated at 4 months. Change in weight was calculated as the difference between the first weight since enrollment—no more than one month after enrollment—and the weight between 12 and 20 weeks that was closest to 4 months from enrollment. Paired *t* tests were used to assess significance in percent weight change at 4 months in the intervention and control groups. To assess if there was a dose-dependent relationship between engagement and weight loss, participants were stratified by engagement cohorts based on number of coaching consults and text messages sent from participant. For each cohort, mean percent weight loss was calculated. The high-engagement cohort was defined as participants who were at the top quartile of messages sent per month or number of coaching consults in the 4-month coaching period. The low-engagement cohort was defined as participants at the bottom quartile of number of messages and video consults. The medium-engagement cohort was defined as participants in the 25th-75th engagement percentiles.

Similarly, baseline blood pressure was designated as the first blood pressure reading since enrollment. Final blood pressure was designated as the blood pressure reading taken between 12 and 20 weeks that was closest to 4 months from enrollment. The mean change in SBP was calculated and assessed for significance using a two-sided *t* test. Furthermore, participants were stratified by hypertension stage—as defined by the Seventh Report of the Joint National Committee on Prevention, Detection, Evaluation, and Treatment of High Blood Pressure (JNC-7)—based on their baseline SBP; this stratification showed the proportion of participants whose final blood pressure placed them in a better or worse stage.

**Table 1:** Baseline demographic summary statistics.

Characteristics		Enrolled participants (N=1012)	Intervention group (n=763)	Matched-pair control group (n=73)	Enrolled versus intervention, P	Intervention versus control, P
Age (years), mean (SD)		44.63 (11.25)	44.78 (11.18)	42.36 (10.28)	.41^a^	.41^a^
Sex (male), n (%)		337 (33.30)	261 (34.2)	17 (23)	.73^b^	.08^b^
Starting BMI^c^ (kg/m^2^), mean (SE)		33.50 (0.21)	33.34 (0.24)	34.33 (0.69)	.59^a^	.19^a^
**BMI distribution (kg/m^2^), n (%)**					.96^b^	.31^b^
	25.0-29.9	368 (36.36)	283 (37.1)	19 (26)		
	30.0-34.9	305 (30.14)	234 (30.7)	26 (36)		
	35.0-39.9	189 (18.67)	138 (18.1)	15 (21)		
	≥40.0	150 (14.82)	108 (14.2)	13 (18)		
**US state, n (%)**					.78^b^	.02^b^
	Colorado	195 (19.27)	155 (20.3)	26 (36)		
	Wisconsin	626 (61.86)	457 (59.9)	36 (49)		
	Georgia	168 (16.60)	136 (17.8)	11 (15)		
	Other	23 (2.27)	15 (2.0)	0 (0)		
**Self-reported comorbidities, n (%)**					.87^b^	.54^b^
	Prediabetes	57 (5.63)	44 (5.8)	4 (5)		
	Diabetes	41 (4.05)	25 (3.3)	0 (0)		
	Hypertension	157 (15.51)	109 (14.3)	7 (10)		
	Hyperlipidemia	124 (12.25)	93 (12.2)	7 (10)		

^a^*P* value of two-sample *t* test for intervention.

^b^*P* value of chi-square test.

^c^BMI: body mass index.

## Results

### Enrollment and Demographics

**Figure 1:**
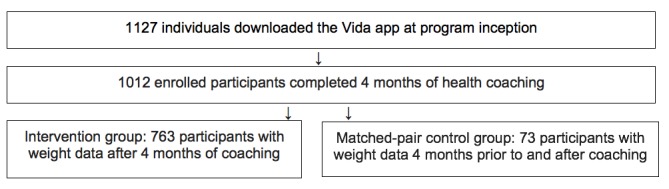
Study population including enrollment and retention.

Participant enrollment and retention in the program is displayed Figure 1. Among the 1127 adults who were initially enrolled in the program, 1012 (89.80%) participants completed 4 months of intensive health coaching at the time of analysis. Of the 1012 enrolled, 763 (75.40%) participants, who will now be referred to as the intervention group, had an initial weight upon enrollment and final weight between 3 and 5 months from enrollment. Of the 1012 enrolled, there were 73 (7.21%) participants who had weight data 4 months prior to and after Vida coaching, who served as the matched-pair control group.

Baseline demographics and chronic disease profiles of all enrolled participants, intervention participants, and matched-pair controls with weight data are reported in Table 1. Of the 1012 total enrolled participants, 337 (33.30%) were men, 368 (36.36%) were overweight, 644 (63.64%) were obese, 98 (9.68%) were prediabetic or diabetic, and 157 (15.51%) had hypertension. These characteristics do not differ significantly between the enrolled participants and the intervention group. Compared to the intervention group, the matched-pair control group had fewer men (261/763, 34.2% intervention vs 17/73, 23% control, *P*=.08) and more obese participants (480/763, 62.9% intervention vs 54/73, 74% control, *P*=.31). These differences were not statistically significant. The only statistically significant difference between the three populations was the distribution of participants in each state between the intervention and matched-pair control groups (*P*=.02).

### Changes in Weight Loss

**Figure 2:**
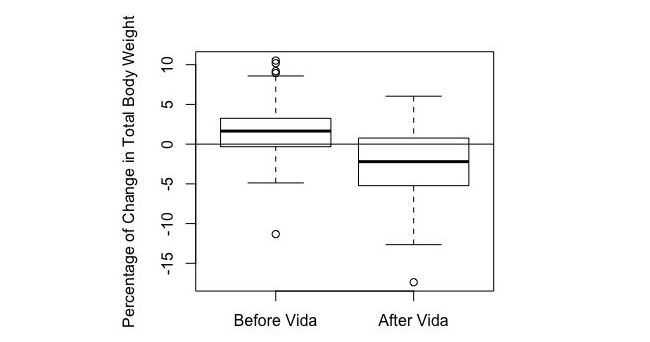
Mean percentage change in weight in matched-pair controls during the 4 months before coaching and 4 months after coaching.

As shown in Table 2, participants in the intervention group lost an average of 3.23% TBW at 4 months of coaching. Out of 763 participants, 218 (28.6%) achieved a clinically significant weight loss of 5% or more of TBW, with a mean of 9.46% weight loss in this cohort. In the matched-pair controls, participants gained an average of 1.81% TBW in 4 months without Vida coaching and lost an average of 2.47% TBW after 4 months of Vida coaching (see Table 3), demonstrating a statistically significant difference of 4.28% in mean percentage weight change (*P*<.001) (see Figure 2).

Figure 2 shows a box plot demonstrating the mean percentage change in weight in controls during the 4 months before Vida coaching and 4 months after Vida coaching. The mean percentage change in body weight and standard error for users before Vida and after Vida are 1.81% (SE 0.41) and -2.47% (SE 0.48), respectively. A two-sided *t* test was performed and demonstrated a statistically significant difference of 4.28% in mean percentage weight change (*P*<.001).

The level of engagement impacted the amount of weight loss among study participants. There were 306 participants in the high-engagement cohort, defined as participants who were at the top quartile of messages sent per month or number of coaching consults in the 4-month coaching period. There were 74 participants in the low-engagement cohort, defined as participants at the bottom quartile of messages and video consults. There were 383 participants in the medium-engagement cohort. The high-engagement cohort lost the most weight, followed by the medium- and then low-engagement cohorts (see Table 4).

**Table 2:** Percentage change from total body weight in intervention group.

Weight and TBW^a^ measures	Weight, mean (SE) or % TBW change (SE)	Number of participants (n=763), n (%)
Baseline weight (kg), mean (SE)	96.23 (0.78)	763 (100)
Weight at 4 months (kg), mean (SE)	92.99 (0.76)	763 (100)
Mean weight loss at 4 months, % TBW change (SE)	-3.23 (0.22)^b^	763 (100)
≥5% weight loss at 4 months, % TBW change (SE)	-9.46 (0.41)^b^	218 (28.6)
≥2% to <5% weight loss at 4 months, % TBW change (SE)	-3.41 (0.06)^b^	210 (27.5)
≥-2% to <2% weight change at 4 months, % TBW change (SE)	-0.30 (0.07)	255 (33.4)
>2% weight gain at 4 months, % TBW change (SE)	+4.90 (0.81)^b^	80 (10.5)

^a^TBW: total body weight.

^b^*P*<.001 for two-sided *t* test.

**Table 3:** Percentage change from total body weight for matched-pair control group before and after Vida program.

Outcome	Weight (n=73), mean (SE) or mean % TBW^a^ change (SE)
Weight 4 months before Vida program (kg), mean (SE)	95.44 (2.26)
Weight at enrollment (kg), mean (SE)	97.10 (2.29)
Weight 4 months after Vida program (kg), mean (SE)	94.65 (2.26)
4 months before Vida program compared to enrollment, mean % TBW change (SE)	1.81 (0.41)^b^
4 months after Vida program compared to enrollment, mean % TBW change (SE)	-2.47 (0.48)^b^

^a^TBW: total body weight.

^b^*P*<.001 for two-sided *t* test.

**Table 4:** Relationship between engagement and weight loss.

Level of cohort engagement	% total body weight loss, mean (SE)	Number of messages per month sent to coach, mean (SE)	Number of consults over 4 months, mean (SE)
Low (n=74)	-1.37 (0.63)	4.83 (2.73)	1.51 (0.20)
Medium (n=383)	-2.84 (0.29)	42.65 (7.14)	5.70 (0.33)
High (n=306)	-3.86 (0.34)	120.59 (12.55)	10.71 (0.26)

### Change in Blood Pressure

**Figure 3:**
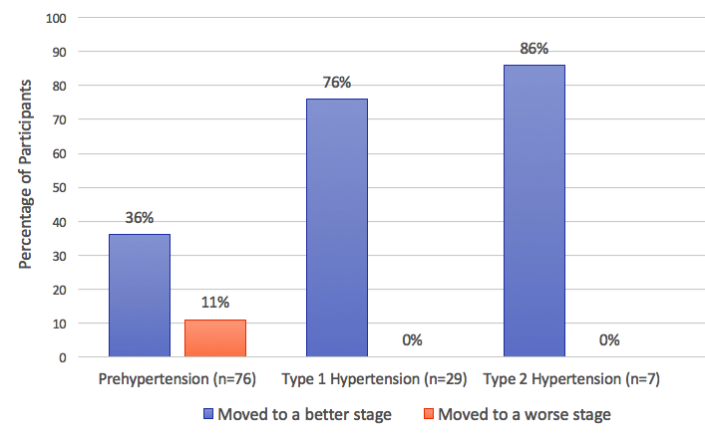
Change in hypertensive category after 4 months of coaching.

Of 151 participants with blood pressure data in Vida, the baseline average SBP was 131 mmHg and the mean change in SBP was a 6 mmHg decrease after 4 months (see Table 5). Among these 151 participants, 112 (74.2%) had a baseline blood pressure that was above the goal, assuming a normal SBP of ≤120 mmHg; 55 out of 112 (49.1%) improved their blood pressure by an entire hypertensive stage—as defined by the JNC-7—at 4 months. Of the 76 users whose baseline SBP was in the prehypertensive range, 27 (36%) had an SBP that was normal and 8 (11%) had an SBP that was in a more severe hypertensive stage after 4 months (see Figure 3). Of the 29 participants with a baseline SBP in the type 1 hypertension range at 4 months, 22 (76%) had an SBP that was in a better stage and none had an SBP in a worse hypertensive stage. Of the 7 participants with a baseline SBP in type 2 hypertension, 6 participants (86%) had a 4-month SBP in a better stage and none had an SBP in a worse stage.

Figure 3 shows the percentage of participants from each hypertension stage at baseline that moved to a better or worse hypertension stage after 4 months of coaching.

**Table 5:** Mean change in systolic blood pressure after 4 months of coaching (n=151).

Outcome	Blood pressure (mmHg), mean (SE)
Baseline SBP^a^	131.27 (1.52)
SBP after 4 months	125.31 (1.18)
Mean change in SBP after 4 months	-5.96 (1.64)^b^

^a^SBP: systolic blood pressure.

^b^*P*=.002 for two-sided *t* test.

### Participant Satisfaction

Of 1012 enrolled participants, 386 (38.14%) submitted ratings of the app, with an average rating of 9.77 out of 10, with 10 being most satisfied (see Table 6). Of the 763 participants in the intervention group, 333 (43.6%) submitted ratings of the app with an average rating of 9.81 out of 10. Mean participant ratings of the app are shown for all available ratings from all enrolled participants and participants from the intervention group.

**Table 6:** Satisfaction data.

Participant cohort	Rating of the app (out of 10), mean (SD)	Participants who provided a rating, n (%)
All enrolled participants (N=1012)	9.77 (0.92)	386 (38.14)
Participants in intervention group (n=763)	9.81 (0.73)	333 (43.6)

## Discussion

In this study, a mobile phone-based health-coaching intervention was found to be effective in reducing weight and blood pressure in overweight and obese adults over a 4-month period, with participants in the matched-pair control group demonstrating a statistically significant difference of 4.28% in mean percentage weight change.

Unlike other interventions targeting more complex conditions [17,26], this program enrolled a broad, geographically diverse, and relatively healthy population intended to mirror the general overweight population of a national commercial payer. Most participants were in their 40s, reflecting the enrollment strategy: a commercially insured population that is, by definition, at least working age but largely younger than Medicare eligibility at 65 years. Similar to other digital health studies with weight loss interventions [17,23-25], our study cohort was predominantly female, which may suggest that women are more likely to engage in weight loss interventions.

Key aspects of the intervention included the ability for participants to self-monitor progress and be accountable for goals set with their health coach. Self-monitoring is the most common feature of mobile apps for health intervention [20,27] and has been shown to have positive effects [25,28,29]. With the ability to log health-related behaviors into the app (eg, food choices and exercise duration) and the ability to see data from wireless pedometers upload instantaneously into the app, participants are able to keep track of their progress as they progress through the intervention. However, with self-monitoring alone, engagement and retention can be a challenge, as most mobile apps have only simplistic capabilities that lack complex user needs and preferences as well as the relationship of a health care expert who can weigh in on their progress [27]. There was high engagement in the study cohort, with 90% of those who downloaded the app completing 4 months of coaching. This is highly important given that failure in clinical interventions for obesity have been primarily attributed to fluctuations in treatment adherence over time, as patients often lack sustained motivation for primary prevention compared to those with more complex conditions who face a more urgent need for behavior change [30,31]. The fact that participants were satisfied with the intervention, given their high rating of the Vida Health app, demonstrates that the mobile app is an effective platform for delivering health coaching. Participants viewed their relationship with their coach favorably, with the limitation that only 44% of those in the intervention group provided feedback.

There was notable weight loss in the intervention group at 4 months, with an average total body weight loss of 3.23%. In addition, 28.6% of participants achieved a clinically significant weight loss of 5% TBW or more, with an average of 9.5% TBW lost in this cohort. The change in weight loss is even more significant when taking into account the fact that the intervention began in late November through the winter holiday season when people have the hardest time losing weight [32]. This compares favorably to a mobile SMS text message-based weight loss program, which produced 3.16% TBW loss in its intervention arm after 4 months [33], and to a mobile phone-based, self-monitoring and health consultation program, which demonstrated 2.86% TBW loss in its intervention arm after 24 weeks [26]. Our results also corroborate findings from Chin et al who demonstrated positive weight loss benefits in their study of the mobile phone, health coach app, Noom, with 77.9% of participants reporting a decrease in body weight during app usage [25].

Unique in this analysis was the availability of 4-month pre- and postcoaching data for a subset of participants that could act as their own matched-pair controls, thereby controlling for the role of intrinsic motivation and allowing the study to isolate the impact of Vida coaching on weight loss. Given that participants in this cohort gained 1.81% TBW over the 4 months prior to using Vida and lost 2.47% TBW over the 4 months after starting Vida suggests the true impact of Vida coaching to be even greater than measured, which is due to the likely continued weight gain in the absence of additional coaching intervention. The dose-dependent relationship between engagement and weight loss further demonstrates that weight loss was driven by engagement with the coach.

In the cohort of 151 participants who had blood pressure data, Vida coaching had a positive impact in reducing average SBP by 5.96 mmHg and by moving significant numbers of participants within each blood pressure stage to a better blood pressure stage after 4 months of coaching. Overall, half of the participants with blood pressure data improved their blood pressure by an entire hypertensive stage after 4 months of coaching, with greater proportions of participants improving at higher stages of baseline hypertension. There is limited data in existing literature regarding the impact of digital health interventions on blood pressure in a comparable cohort. One meta-analysis found that digital health interventions did not have statistically significant impact on blood pressure [34]. Willey et al demonstrated a mean SBP change of 18.6 mmHg after 12 weeks of a digital health app intervention that delivered guided nutritional and physical exercise plans, albeit the sample size was limited to 10 participants [35].

There were limitations to this study. Criteria for enrollment were largely self-reported. Participants skewed toward having more female than male participants and toward being a relatively young cohort, making generalization to older individuals more difficult. While participants were geographically diverse, it is challenging to draw conclusions around the impact of the intervention on minority populations or populations of varying education and income brackets, given the lack of demographic data around ethnicity and socioeconomic status. All participants were mobile phone owners, which is associated with higher socioeconomic status and, therefore, possibly less prevalence of obesity and chronic disease [36,37]. However, mobile phone ownership has high penetrance, with the majority of people owning mobile phones even in the lowest education and income brackets [37]. Another limitation was the lack of a true control group, though this was addressed by using the closest proxy available with participants who had weight data prior to receiving coaching, albeit a small sample size. The study was also limited by data availability and attrition. A total of 15% of those enrolled completed the program but could not be included in the analysis due to lack of weight data. Technical challenges with hardware devices likely contributed to lack of weight data, given the large number of technical assistance requests from participants regarding their pedometers and scales. Further analyses of clinical results and cost-effectiveness at future time points are needed.

Despite these limitations, this study demonstrates that a mobile app-based coaching intervention can be an acceptable and effective means to achieving desired clinical results for overweight and obese individuals. As mobile phones continue to penetrate the consumer market, digital health coaching may serve as a promising model to increase access to evidence-based behavioral coaching for obesity and related cardiovascular conditions.

## Abbreviations

BMI: body mass index
IRB: Institutional Review Board
JNC-7: Seventh Report of the Joint National Committee on Prevention, Detection, Evaluation, and Treatment of
SBP: systolic blood pressure
SMS: short message service
TBW: total body weight
UCSF: University of California, San Francisco
